# Self-Management, Adherence, and the Role of Pharmaceutical Care in Patients with T2DM in Primary Practice: A Cross-Sectional Survey in Bulgaria

**DOI:** 10.3390/pharmacy14010035

**Published:** 2026-02-12

**Authors:** Petya Milushewa, Nataliya Chenesheva, Valentina Petkova

**Affiliations:** Faculty of Pharmacy, Medical University of Sofia, 1000 Sofia, Bulgaria; p.milusheva@pharmfac.mu-sofia.bg (P.M.); 105374@students.mu-sofia.bg (N.C.)

**Keywords:** T2DM, pharmaceutical care, patient education, medication adherence, self-management, community pharmacy, pharmacist-led interventions

## Abstract

Background: Type 2 diabetes mellitus (T2DM) is a prevalent chronic disease requiring effective pharmacological treatment, sustained self-management, and patient education. Pharmacists are increasingly recognized as key contributors to diabetes care; however, their role remains underutilized in Bulgaria. This study aimed to assess self-management behaviors, medication adherence, patient awareness, and the perceived role of pharmacists among patients with T2DM in Bulgarian primary care. Methods: A cross-sectional observational study was conducted among 105 patients with T2DM using an anonymous questionnaire based on the Diabetes Self-Management Questionnaire and supplementary items adapted to the local healthcare context. Data were analyzed using descriptive statistics and non-parametric tests to explore associations between demographic characteristics, treatment patterns, self-management behaviors, and educational needs. Results: Most patients were treated with oral antidiabetic therapy (90.0%), predominantly metformin-based regimens (64.0%). Adherence to prescribed pharmacological treatment was high (93.0%), while adherence to dietary recommendations (70.0%), regular physical activity (60.0%), and blood glucose self-monitoring (63.0%) was less consistent. Although 92.0% of participants reported good or excellent disease awareness, 41.0% expressed a need for additional education, particularly regarding confidence in managing hypoglycemia and the use of digital monitoring tools. More than half of respondents (54.0%) had received diabetes-related information from a pharmacist; however, only 38.0% expressed willingness to participate in pharmacist-led education, while 34.0% were undecided. Female sex was associated with a higher prevalence of comorbidities (*p* = 0.010), while increasing age was associated with reduced metformin use (*p* = 0.004). Conclusions: Despite good pharmacological adherence and self-reported awareness, gaps remain in lifestyle-related self-management and patient education. The findings support an expanded role for pharmacists in diabetes care, particularly through structured educational and counseling interventions to enhance self-management and complement physician-led treatment.

## 1. Introduction

Type 2 diabetes mellitus (T2DM) is a multifactorial chronic disease and a major and growing public health burden worldwide. Characterized by insulin resistance and progressive pancreatic β-cell dysfunction, T2DM is the most prevalent form of diabetes, accounting for over 90% of all diagnosed cases [1]. In 2024, over 95% of the 589 million adults living with diabetes globally were affected by type 2 diabetes mellitus (T2DM), corresponding to an estimated global T2DM prevalence of approximately 11%. In Europe, T2DM accounted for the vast majority of the 66 million adults with diabetes (prevalence 9.8%), with a higher burden observed in Eastern Europe and the Balkans, where prevalence exceeded 10% in several countries [2]. This alarming trend is tightly linked to the global rise in obesity, sedentary lifestyles, and population aging. T2DM is not merely an isolated metabolic condition; rather, it is often the terminal expression of a broader metabolic dysregulation known as metabolic syndrome [3]. This syndrome comprises abdominal obesity, dyslipidemia, hypertension, and impaired glucose tolerance, which together substantially increase cardiovascular morbidity and mortality [4,5].

In recent years, therapeutic paradigms in diabetes care have shifted from purely glycemic control toward a more holistic, patient-centered model. While lifestyle modifications such as moderate weight loss (5–15%) and increased physical activity remain foundational, many patients continue to require pharmacological interventions to maintain long-term control. Contemporary guidelines emphasize the importance of individualized treatment plans, incorporating agents such as GLP-1 receptor agonists and SGLT-2 inhibitors for their demonstrated cardio-renal benefits, especially in patients with comorbidities [6,7]. However, optimal treatment outcomes depend not only on drug efficacy but also on patients’ adherence, self-monitoring behaviors, and health literacy. Suboptimal adherence is associated with poor glycemic control, increased complication risk, and higher healthcare costs [8]. In this context, pharmaceutical care, defined as the responsible provision of drug therapy to achieve specific outcomes that improve a patient’s quality of life, has emerged as a critical component of chronic disease management [9]. Pharmacists, due to their accessibility and expertise, are uniquely positioned to support patients with type 2 diabetes through education, medication review, monitoring, and self-care counseling. Recent systematic reviews and meta-analyses show that pharmacist-led interventions are associated with significant improvements in clinical outcomes such as reductions in HbA1c, systolic blood pressure, LDL cholesterol, and enhanced medication adherence in T2DM patients [10,11].

Despite global efforts to standardize evidence-based care for T2DM, significant disparities persist in how diabetes management is delivered and experienced across health systems, particularly in Central and Eastern Europe. In Bulgaria, diabetes care remains largely physician-centered and is characterized by fragmented care pathways, limited access to structured diabetes education, and minimal involvement of community pharmacists in chronic disease management. Pharmacists’ roles are predominantly restricted to medication dispensing, with limited formal engagement in patient counseling, adherence monitoring, or structured educational programs [12].

This study aims to assess self-management practices, medication adherence, and informational support among patients with T2DM in Bulgarian primary care, with a special focus on the perceived role of pharmacists and patients’ interest in participating in pharmacist-led educational and counseling programs.

## 2. Materials and Methods

A cross-sectional observational study was conducted between June and August 2025 in a primary care setting in Bulgaria, using an anonymous online questionnaire distributed among patients diagnosed with T2DM who were followed at the practice of a general practitioner. Participants were adult patients (≥18 years) with a confirmed diagnosis of T2DM who were receiving regular follow-up care at the general practitioner’s practice during the study period. All eligible patients attending the general practitioner’s practice during the study period were systematically approached and invited to participate. Participation was voluntary, and all respondents completed the questionnaire anonymously. Recruitment continued consecutively until the predetermined target sample size was achieved. Given the exploratory nature of the study and its primary focus on descriptive outcomes (self-management practices, medication adherence, and informational support), a minimum sample size of approximately 100 participants was considered sufficient to provide reliable prevalence estimates with acceptable precision and to allow for exploratory subgroup comparisons using non-parametric statistical methods.

The survey instrument was developed based on the validated Diabetes Self-Management Questionnaire (DSMQ) and was further supplemented with items adapted from educational materials published by Diabetes UK, as well as additional questions specifically designed to reflect the local healthcare context and the role of the pharmacist in diabetes management. The final questionnaire consisted of four main sections: demographic characteristics; pharmacotherapy; self-management and self-monitoring; patient awareness and support. Examples of locally adapted items included: use of community pharmacy services for diabetes-related advice, willingness to participate in pharmacist-led educational programs, perceived need for additional diabetes education, and use of digital monitoring tools. Items were rated on a four-point Likert scale (“does not apply”, “rather does not apply”, “rather applies”, “fully applies”). For analysis, responses were grouped into three categories: “rather/fully applies”, “does not apply”, and “not sure”. Negatively worded items were reverse-coded so that higher values consistently reflected better self-management. DSMQ-derived variables were analysed at the domain level, including glucose monitoring, dietary adherence, medication adherence, physical activity, confidence in hypoglycemia management, healthcare communication, guideline-based self-management, adherence barriers, and use of digital monitoring tools. Results were presented as absolute and relative frequencies and interpreted descriptively to identify behavioral domains requiring targeted educational interventions.

Collected data were coded and analyzed using IBM SPSS Statistics, version 29. Descriptive statistics were applied to summarize the study variables. Categorical variables were presented as absolute frequencies (*n*) and relative frequencies (%). Continuous variables were described using median (Me) and interquartile range (IQR); the arithmetic mean was additionally reported for completeness. Associations between categorical variables were assessed using the Chi-square test, with Fisher’s exact test applied where appropriate. Comparisons between two independent groups were performed using the Mann–Whitney U test, while comparisons among more than two groups were conducted using the Kruskal–Wallis test. A *p*-value of <0.05 was considered statistically significant.

## 3. Results

### 3.1. Participant Characteristics

A total of 105 patients with type 2 diabetes mellitus (T2DM) were included in this study. The majority of participants were aged between 50 and 69 years, with the highest proportion observed in the 50–59 and 60–69 age groups. Female participants predominated, accounting for 58% of the study population. The median duration of diabetes was 8 years (IQR: 3–12). Detailed demographic characteristics of the participants are presented in Table 1.

### 3.2. Antidiabetic Therapy

Among the total study population (*n* = 105), the majority of patients were receiving pharmacological treatment for type 2 diabetes mellitus. Oral antidiabetic therapy was reported by 95 patients (90.0%), while 1 patient (1.0%) was treated with insulin alone and 2 patients (2.0%) received combined therapy with oral agents and insulin. A smaller proportion of participants (7 patients, 7.0%) reported managing their condition exclusively through diet and physical activity. Further analysis of antidiabetic medication classes among patients receiving pharmacological treatment (*n* = 98) revealed that metformin-based regimens were the most commonly prescribed, reported by 63 patients (64.0%). The distribution of antidiabetic therapy modalities and medication classes is summarized in Table 2.

### 3.3. Self-Management and Self-Monitoring Behaviors

Regular blood glucose self-monitoring was reported by 62.9% of participants, whereas 31.4% indicated irregular or insufficient monitoring. Adherence to dietary recommendations was reported by 69.5%, while adherence to prescribed pharmacological therapy was high (93.3%). Most patients (93.3%) reported discussing their treatment with a physician or pharmacist when questions arise. Engagement in regular physical activity was reported by 60.0% of respondents. Confidence in managing hypoglycemic episodes was observed in 64.8%, although 24.7% remained uncertain. Difficulties related to treatment adherence were uncommon, reported by 16.2% of participants. The majority (83.8%) reported making efforts to self-manage their condition in accordance with professional recommendations. Nevertheless, 41.0% expressed a need for additional education regarding diabetes and its treatment. Use of digital tools for diabetes monitoring was limited (22.9%). A summary of self-management and adherence behaviors is presented in Table 3.

### 3.4. Patient Awareness, Pharmacist-Provided Information, and Educational Needs

The majority of participants (92.0%) reported a good or excellent level of awareness regarding their diabetes and its treatment. More than half of the respondents (54.0%) indicated that they had received diabetes-related information or advice from a pharmacist, either during medication dispensation or following a specific inquiry. With regard to pharmacist-led educational activities, 38.0% of patients expressed willingness to participate in short training sessions or consultations, while 34.0% remained undecided. Additionally, 41.0% of respondents reported a perceived need for further education related to diabetes and its management. Among the educational topics proposed, nutrition and diet were most frequently selected (32.0%), followed by diabetes complications and their prevention, self-monitoring and digital technologies, and pharmacological therapy. A summary of patient awareness, pharmacist involvement, and educational needs is presented in Table 4.

### 3.5. Statistical Analysis of Self-Management and Educational Needs

Table 5 summarizes the statistically significant and clinically relevant associations between selected demographic characteristics and parameters related to antidiabetic therapy, comorbidities, and educational needs. A significant association was observed between sex and the presence of chronic comorbidities, with female patients reporting a higher prevalence of concomitant chronic conditions compared to male patients (*p* = 0.010). In contrast, no statistically significant sex-related differences were identified with regard to antidiabetic treatment type, use of specific medication classes, receipt of pharmacist-provided information, or willingness to participate in pharmacist-led educational activities. Age-related differences were observed for selected treatment and educational parameters. Metformin use decreased significantly with increasing age, with patients aged 70 years and older being less frequently prescribed metformin compared to younger age groups (*p* = 0.004). In addition, the prevalence of chronic comorbidities showed a progressive increase across age groups, approaching statistical significance (*p* = 0.051). With respect to educational preferences, a significant decline in interest in psychological support and motivational counseling was observed with increasing age (*p* = 0.020). No statistically significant associations were found between age groups and willingness to participate in pharmacist-led education, receipt of information from a pharmacist, or interest in other educational topics. Overall, Table 5 shows that age and sex influence selected clinical and educational aspects of diabetes management, whereas most parameters related to pharmacist involvement and patient education are comparable across demographic groups.

## 4. Discussion

The present study provides insight into self-management behaviors, treatment adherence, patient awareness, and the potential role of pharmacists among patients with T2DM in primary care. The findings highlight generally good adherence to pharmacological therapy and high self-reported disease awareness, alongside notable gaps in lifestyle management, confidence in hypoglycemia management, and utilization of pharmacist-led educational support.

The predominance of oral antidiabetic therapy and the widespread use of metformin observed in this study are consistent with current international recommendations, which continue to position metformin as first-line therapy in patients without contraindications or high cardiovascular risk [13,14]. High levels of self-reported adherence to prescribed therapy are comparable to findings from previous European studies, suggesting that medication-taking behavior may be better preserved than lifestyle-related self-management components in patients with T2DM [15]. Age-related differences in metformin use, particularly the lower prevalence among patients aged 70 years and older, may reflect concerns related to renal function, gastrointestinal tolerability, or polypharmacy in older adults [14]. These findings underscore the importance of individualized treatment strategies in elderly patients, an area where pharmacist-led medication review may offer added value.

Despite good pharmacological adherence, suboptimal engagement in regular physical activity and inconsistent self-monitoring of blood glucose were observed in a substantial proportion of participants. This pattern aligns with previous research indicating that lifestyle modification remains one of the most challenging aspects of diabetes management [15,16]. Socioeconomic barriers, cultural attitudes toward physical activity, limited access to structured lifestyle education programs, and time constraints have been identified as major obstacles to sustained lifestyle change in patients with T2DM, both globally and in Eastern European settings [17,18]. In Bulgaria, the limited availability of multidisciplinary diabetes education services and the absence of structured community-based lifestyle programs may further contribute to these gaps.

The observed uncertainty regarding appropriate responses to hypoglycemic episodes further emphasizes the need for structured patient education. In the present study, 25% of participants reported uncertainty regarding hypoglycemia management, which may reflect insufficient education, fear of hypoglycemic events, or limited access to continuous professional support. Similar findings have been reported in previous studies, where inadequate patient training and low self-efficacy were associated with impaired hypoglycaemia recognition and response [16]. Targeted educational programs focusing on symptom recognition, prevention strategies, and practical management algorithms are therefore warranted.

The progressive increase in comorbid conditions with age, particularly among female participants, mirrors epidemiological data linking T2DM with multimorbidity and metabolic syndrome [19]. These findings reinforce the need for integrated, multidisciplinary care approaches targeting both glycemic control and associated cardiovascular risk factors.

Although most participants rated their awareness of diabetes and its treatment as good or excellent, a considerable proportion expressed a perceived need for additional education. This apparent discrepancy between self-assessed knowledge and expressed educational needs has been reported previously and may reflect partial understanding of disease complexity rather than true mastery of self-management skills [20]. Previous studies have shown that patients often overestimate their level of disease knowledge while still lacking practical competencies required for effective self-care, particularly in areas such as diet planning, physical activity, and glucose monitoring [15]. This phenomenon has been described as a “knowledge–practice gap” in diabetes self-management [16].

Nutrition and diet were identified as the most desired educational topics, followed by diabetes complications and self-monitoring technologies. These preferences are consistent with international studies highlighting patient demand for practical, lifestyle-oriented guidance rather than exclusively pharmacological information [21].

More than half of the participants reported having received diabetes-related information from a pharmacist; however, a substantial proportion had not engaged in such interactions. This finding suggests that pharmacist involvement in diabetes care remains underutilized, despite growing evidence supporting its effectiveness. Multiple randomized trials and meta-analyses have demonstrated that pharmacist-led interventions can significantly improve glycemic control, medication adherence, and cardiovascular risk profiles in patients with T2DM [22,23,24]. In addition, pharmacist-delivered education has been shown to enhance patient confidence, self-efficacy, and long-term engagement in self-management behaviors [25].

The willingness or uncertainty expressed by most participants regarding participation in pharmacist-led educational initiatives indicates a clear opportunity for expanding pharmaceutical care services within community and primary care settings. Structured educational programs, individualized counseling, and digital self-monitoring support represent feasible and evidence-based strategies for improving diabetes outcomes. Structured pharmacist-led education programs, individualized counselling, and digital self-monitoring support represent feasible and evidence-based strategies for addressing the identified gaps in lifestyle management and hypoglycemia confidence.

The findings of this study support the integration of pharmacists as active members of the diabetes care team, particularly in areas related to patient education, medication review, and self-management support. However, the cross-sectional design and reliance on self-reported data limit causal interpretation and may introduce reporting bias. Additionally, the relatively small sample size and single-setting recruitment may affect generalizability. Future research should explore longitudinal outcomes of pharmacist-led interventions and assess their impact on objective clinical endpoints, including HbA1c, cardiovascular risk markers, and healthcare utilization.

## 5. Conclusions

This study explored antidiabetic treatment patterns, self-management behaviors, patient awareness, and pharmacist involvement among patients with T2DM. Pharmacological adherence and self-reported disease awareness were generally high, with metformin-based oral therapy predominating. In contrast, lifestyle-related self-management practices and confidence in hypoglycemia management were less consistent. Selected demographic differences were identified. Female patients reported a higher burden of chronic comorbidities, while increasing age was associated with reduced metformin use and lower interest in psychological and motivational support. Despite high perceived awareness, a substantial proportion of patients expressed a need for additional education, particularly in relation to nutrition, diabetes complications, and self-monitoring technologies. Overall, the findings support the expanded role of pharmacists in diabetes care, particularly in patient education, medication review, and self-management support. Integrating structured pharmacist-led interventions into routine care may address existing gaps and complement physician-led management. Further studies are warranted to evaluate the long-term clinical impact of such models in broader patient populations.

## Figures and Tables

**Table 1:** Demographic characteristics of the study population (*n* = 105).

Characteristic	*n*	%
Sex		
Male	44	42.0
Female	61	58.0
Age group (years)		
<40	2	2.0
40–49	17	16.0
50–59	32	30.0
60–69	29	28.0
70–79	20	19.0
≥80	5	5.0
Duration of T2DM		
<1 year	11	10.0
1–5 years	24	23.0
6–10 years	28	27.0
>10 years	42	40.0

**Table 2:** Antidiabetic therapy patterns among the study population.

Variable	*n*	%
Current diabetes management (total sample, *n* = 105)		
Diet and physical activity only	7	7.0
Oral antidiabetic therapy	95	90.0
Insulin therapy	1	1.0
Combination therapy (oral agents + insulin)	2	2.0
Antidiabetic medication classes * (*n* = 98)		
Metformin (biguanides)	63	64.0
Sulfonylureas	11	11.0
GLP-1 receptor agonists	10	10.0
SGLT-2 inhibitors	7	7.0
Insulin	3	3.0
DPP-4 inhibitors	2	2.0
Other antidiabetic agents	4	4.0

* Percentages for medication classes are calculated among patients receiving pharmacological treatment (*n* = 98) and may exceed 100% due to combination therapy.

**Table 3:** Summary of self-management and adherence behaviors among patients with type 2 diabetes mellitus (*n* = 105).

Behavior	Rather/Fully Applies *n* (%)	Does Not Apply *n* (%)	Not Sure *n* (%)
Regular blood glucose self-monitoring	66 (62.9)	33 (31.4)	6 (5.7)
Adherence to dietary recommendations	73 (69.5)	26 (24.8)	6 (5.7)
Adherence to prescribed therapy	98 (93.3)	3 (2.9)	4 (3.8)
Discussion with physician or pharmacist	98 (93.3)	6 (5.7)	1 (1.0)
Regular physical activity	63 (60.0)	37 (35.2)	5 (4.8)
Confidence in managing hypoglycaemia	68 (64.8)	11 (10.5)	26 (24.7)
Difficulties with treatment adherence	17 (16.2)	84 (80.0)	4 (3.8)
Efforts toward guideline-based self-management	88 (83.8)	16 (15.2)	1 (1.0)
Need for additional diabetes education	43 (41.0)	51 (48.6)	11 (10.4)
Use of digital monitoring tools	24 (22.9)	75 (71.4)	6 (5.7)

“Rather applies” and “Fully applies” responses were pooled. Percentages are based on the total sample (*n* = 105) and rounded to one decimal place. Absolute frequencies sum to 105 for all variables.

**Table 4:** Patient awareness, pharmacist involvement, and educational needs (summary) (*n* = 105).

Parameter	*n* (%)
Good or excellent self-reported awareness of diabetes	96 (92.0)
Received diabetes-related information from a pharmacist	57 (54.0)
Willing to participate in pharmacist-led education	40 (38.0)
Undecided about pharmacist-led education	36 (34.0)
Perceived need for additional diabetes education	43 (41.0)
Most requested educational topic: nutrition and diet	73 (69.5) *

* Multiple responses allowed.

**Table 5:** Statistically significant associations between demographic characteristics and treatment- and education-related parameters.

Demographic Characteristic	Outcome Variable	Direction of Association	Statistical Test	*p*-Value
Sex	Presence of chronic comorbidities	Higher prevalence in women	Chi-square test	0.010
Age group	Metformin use	Lower use in patients ≥70 years	Kruskal–Wallis test	0.004
Age group	Presence of chronic comorbidities	Increasing prevalence with age	Kruskal–Wallis test	0.051
Age group	Interest in psychological support and motivation	Decreasing interest with age	Kruskal–Wallis test	0.020

## Data Availability

The data presented in this study are available on request from the corresponding author due to ethical restrictions, as they were collected through an anonymous questionnaire and contain sensitive information that could compromise participant privacy if disclosed.
